# Beneficial role of Coronatine on the morphological and physiological responses of Cress Plants (*Lepidium sativum*) exposed to Silver Nanoparticle

**DOI:** 10.1186/s40529-024-00425-z

**Published:** 2024-07-10

**Authors:** Shahla Hashemi Shahraki, Fereshteh Mohamadhasani Javar, Babak Jamali, Fatemeh sargazi

**Affiliations:** 1 Biology Department, Faculty of Science, University of Sistan and Baluchestan, Zahedan, Iran; 2 Department of Biology, Faculty of Science, Payame Noor University (PNU), Tehran, Iran; 3 Department of Agriculture, Minab Higher Education Center, University of Hormozgan, Bandar Abbas, Iran

**Keywords:** Silver oxide, Cress, Coronatine, Nanoparticles

## Abstract

**Background:**

Silver nanoparticles are widely used in various fields such as industry, medicine, biotechnology, and agriculture. However, the inevitable release of these nanoparticles into the environment poses potential risks to ecosystems and may affect plant productivity. Coronatine is one of the newly identified compounds known for its beneficial influence on enhancing plant resilience against various stress factors. To evaluate the effectiveness of coronatine pretreatment in mitigating the stress induced by silver nanoparticles on cress plants, the present study was carried out.

**Results:**

Our findings indicated a decrease in multiple growth parameters, proline content, chlorophyll a, chlorophyll b, total chlorophyll, and carotenoids in cress plants exposed to silver nanoparticle treatment. This decline could be attributed to the oxidative stress induced by the presence of silver nanoparticles in the plants. Conversely, when coronatine treatment was applied, it effectively mitigated the reduction in growth parameters and pigments induced by the silver nanoparticles. Furthermore, we observed an increase in silver content in both the roots and shoot portions, along with elevated levels of malondialdehyde (MDA) content, hydrogen peroxide (H_2_O_2_), anthocyanins, glutathione (GSH), and antioxidant enzyme activities in plants exposed to silver nanoparticles. Concurrently, there was a decrease in total phenolic compounds, ascorbate, anthocyanins, and proline content. Pre-treatment of cress seeds with coronatine resulted in increased levels of GSH, total phenolic compounds, and proline content while reducing the silver content in both the root and shoot parts of the plant.

**Conclusions:**

Coronatine pre-treatment appeared to enhance both enzymatic and non-enzymatic antioxidant activities, thereby alleviating oxidative stress and improving the response to stress induced by silver nanoparticles.

## Background

Nanoparticles, typically ranging in size from 1 to 100 nanometers, have garnered significant attention and raised concerns due to their widespread application across various industries (Khot et al. 2012; Nair et al. 2010; Hashemi et al. 2016a, b; Sadeghi et al. 2022). The widespread use of nanoparticles across diverse industrial sectors has made their introduction into agricultural water sources inevitable, potentially affecting various agricultural crops. However, excessive levels of nanoparticle release into the environment can lead to their accumulation in living organisms, including plants and animals, thereby adversely influencing plant productivity and human health (Hashemi et al. 2019). Under controlled conditions, e.g., at low concentrations, nanoparticles containing essential nutrients can be beneficial for plant performance. Nevertheless, once these nanoparticles exceed a certain concentration, they lead to toxicity (Usman et al. 2020; Hashemi et al. 2016a, b; Tohidiyan et al. 2019).

Certain reports have highlighted the inhibitory impact of oxidative stress on critical plant developmental stages such as germination and root elongation (Lin and Xing 2007). Boonyanitipong et al. (2011) studied 10 to 1000 ppm of ZnO and titanium dioxide nanoparticles, focusing on rice seed germination (Boonyanitipong et al. 2011). They reported that none of the nanoparticles caused a reduction in germination percentages. Nevertheless, ZnO nanoparticles resulted in reduced root elongation and decreased root volume, while titanium dioxide nanoparticles did not exhibit inhibitory effects on root elongation.

Ahmed et al. (2022) studied the toxic effects of Al_2_O_3_ nanoparticles on maize seedlings. Their findings revealed that Al_2_O_3_ nanoparticles accumulated in both the roots and shoots of the plants, triggering oxidative stress, which subsequently led to a reduction in chlorophyll and protein synthesis, nutrient content, and overall plant growth (Ahmed et al. 2022). Among various nanoparticles, silver nanoparticles have garnered significant attention due to their noteworthy physicochemical properties and wide-ranging applications (Ejaz et al. 2018; Hussain et al. 2019). Multiple reports suggest that an optimal concentration of silver nanoparticles can promote plant growth, boost seed germination, and enhance chlorophyll content and photosynthetic efficiency (Tymoszuk 2021; Aqeel et al. 2022; Gupta et al. 2018).

Exposure to silver nanoparticles in appropriate concentrations has been shown to stimulate plant growth, while both low and high concentrations have detrimental effects (Geisler-Lee et al. 2013; Pereira et al. 2018). In a study by Cvjetko et al. (2017), they observed that silver nanoparticles led to increased silver uptake in the roots of *Allium cepa* plants, resulting in a significant reduction in root growth and oxidative damage (Cvjetko et al. 2017). Numerous studies have revealed that silver nanoparticles can induce physiological and molecular effects on plants, including disruptions in photosynthetic efficiency, inhibition of root growth, induction of cellular oxidative stress, reduction in biomass, and damage to genetic material (Yan and Chen 2019; Siddiqi and Husen 2022; Sami et al. 2020).

Yin et al. (2012) reported that different plant species exhibit varying sensitivities to exposure to silver nanoparticles (Yin et al. 2012).

*Lepidium sativum* L., commonly known as garden cress, is a small, herbaceous, annual plant that reaches a height of about 50 centimeters. It belongs to the brassicaceae family and is renowned for its medicinal properties, used for the treatment of inflammation, bronchitis, blood purification, vasodilation, and scurvy (Rehman et al. 2012). Due to its therapeutic and nutritional characteristics, cress is cultivated extensively worldwide (Sharma and Agarwal 2011). Environmental changes are a source of stress that plants need to adapt to (Sharma and Agarwal 2011).

Cress plant can be grown and harvested in various climates and soil conditions throughout the year. Its short growth period and high sensitivity to minor environmental changes make it suitable for studying environmental stressors (Smolinska and Szczodrowska 2017; Babaei et al. 2022).

Previous studies have explored the impacts of salinity (Babaei et al. 2022), heavy metal (Nouri and Haddioui 2021), and heat (El-Sattar et al. 2024) stress on the growth and development of cress plants. However, to date, there have been no reports on the effects of nanoparticles on cress plants. Recently, bio-stimulators have gained attention for their potential to enhance plants tolerance to both biotic and abiotic stresses (Zulfiqar et al. 2023; Ma et al. 2022; Hosseinifard et al. 2022).

**Fig. 1:**
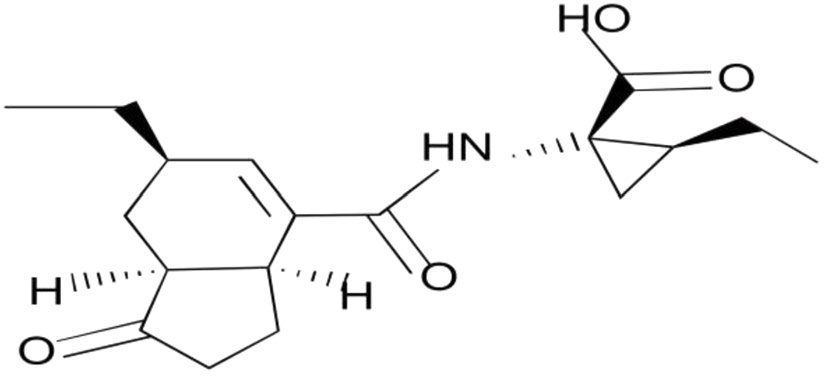
Chemical structure of coronatine

Researchers have identified certain compounds produced by bacteria and fungi, which, owing to their influence on plant growth, are categorized as bio-stimulators. Among these compounds, coronatine stands out as a phytoxin that plays a role in enhancing plant resilience to stress by augmenting the activation of the defense system (Uppalapati et al. 2005) (Fig. 1). Studies point to coronatine as a promising agent that offers protective effects against a spectrum of environmental stressors, encompassing heat, drought, salinity, and heavy metal exposure (CEYLAN et al. 2023; Xie et al. 2008; Wang et al. 2009). A variety of reports demonstrate that coronatine also plays a pivotal role in orchestrating plant responses to stressors induced by pathogens and herbivores, highlighting its importance as a mediator of plant responses to environmental challenges. While the efficacy of coronatine in ameliorating various environmental stressors such as salinity, drought, cold, and frost has been investigated in some plant species, studies on its potential to mitigate nanoparticle-induced stress have been largely lacking.

Given the prevalent use of silver nanoparticles across various nanoparticle types and the limited documentation on their impact alongside bio-stimulants on cress plants, our study aimed to assess the efficacy of coronatine treatment in mitigating the stress induced by silver nanoparticles in cress plants.

## Materials and methods

### Coronatine and silver nanoparticles treatments

**Fig. 2:**
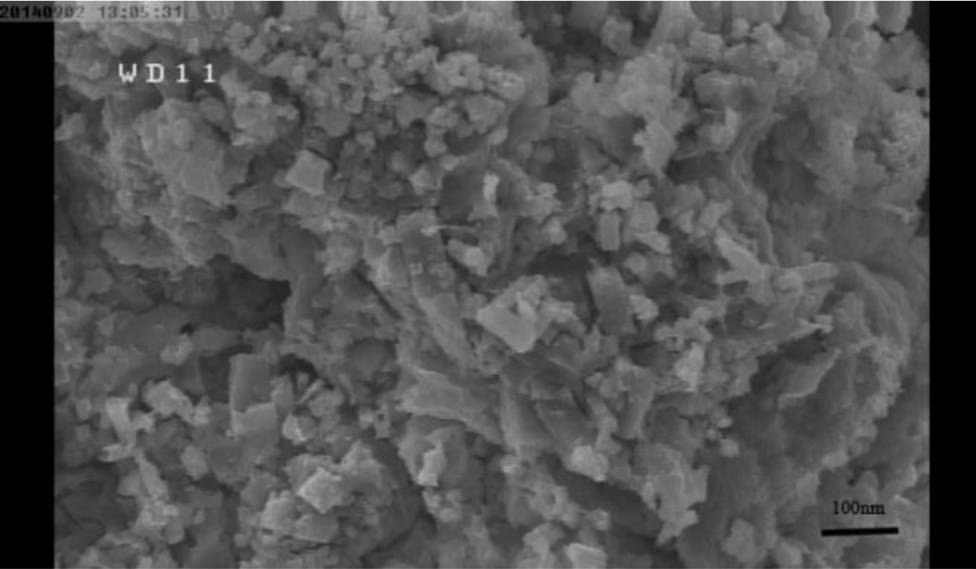
Scanning electron microscope image of the silver nanoparticles

To determine the toxic concentrations of silver nanoparticles and the effective concentration of coronatine on the growth parameters of cress plants, preliminary experiments were carried out. In these experiments, the effects of different concentrations of silver nanoparticles (0, 40, 60, 80, and 100 ppm) and coronatine (0, 60, 80, and 100 nM) on the fresh and dry weight of roots and shoots of 10-day-old seedlings were investigated. Based on the findings of the preliminary experiments (Data not shown, figure suppl number 1–8), concentrations of 80 and 100 ppm silver nanoparticles and 80 nM coronatine were selected for the main experiment. The main experiment was conducted in a completely randomized design with three replications. The cress seeds were first surface-disinfected with 5% sodium hypochlorite for one minute and then rinsed three times with distilled water. Subsequently, these surface-disinfected seeds were placed in petri dishes lined with filter paper and exposed to coronatine treatment at concentrations of 0 or 80 nM for 72 h. The seedlings were then transplanted into pots filled with perlite and transferred to a greenhouse environment with the following prevailing conditions: temperature at 25 °C, relative humidity at 50%, a day/night photoperiod of 16 h light and 8 h dark, and a light intensity of 120 µmol m^− 2^ s^− 1^. During the initial week, the seedlings were irrigated with a ½ hoagland nutrient solution. Subsequently, for a period of 14 days, the seedlings received irrigation every 3 days with a complete hoagland solution, which included various concentrations of silver nanoparticles (0, 80, and 100 ppm). The purchased silver nanoparticles, utilized in powder form, had an average reported size of 20 nanometers according to the manufacturer’s information (US Research Nanomaterial, Inc., 3302 Twig Leaf Lane, Houston, TX 77,084, USA). The size of nanoparticles was confirmed by scanning electron microscope (Model JSM 6390LV, JOEL, USA) and showed that the nanoparticles were homogeneous (Shahraki et al. 2024). Following this experimental phase, the plants were harvested for further evaluation. Following this experimental phase, the plants were harvested for further evaluation Fig. 2.

### Growth parameters

The plants were removed from the pots and, after careful washing with distilled water, the shoot and root segments were gently separated. The length of the shoots and roots was then measured with a ruler.

### Chlorophyll and carotenoid concentration

The leaf discs, each weighing 0.5 g, were extracted in 5 ml of 80% acetone and then centrifuged at 8,000 × g for 10 min. The resulting supernatant was then diluted to a final volume of 100 ml for the leaf extract. The extraction was repeated until decolorization was achieved. The absorbance of the extract was measured at 470, 645, and 663 nm using a spectrophotometer with 80% acetone as a blank. The chlorophyll and carotenoid contents were then calculated using the following equations: (Lichtenthaler 1987).

Chl a (mg. g^− 1^ fresh weight): [(12.25A663–2.79A645) × v / 1000 × W]

Chl b (mg. g^− 1^ fresh weight): [(21.50A645- 5.10A663) × v / 1000 × W]

Carotenoids (mg. g^− 1^ fresh weight): 1000A470- 1.82Chla- 85.02Chlb / 198.

Chla + Chlb (mg. g^− 1^ fresh weight): [(7.15A663 + 18.71A645) × v /1000 × W]

where Chla = chlorophyll a; Chlb = chlorophyll b; Chla + b = total chlorophyll; A = absorbance at ƛ (nm).

### Lipid peroxidation and hydrogen peroxide (H_2_O_2_)

To assess membrane lipid peroxidation, the concentration of malondialdehyde (MDA), a byproduct of the peroxidation of unsaturated fatty acids, was quantified. The MDA measurement followed the method described by Heath and Packer (1969). Initially, 0.2 g of freeze-dried leaf tissue was weighed and homogenized in 5 ml of 0.1% trichloroacetic acid (TCA) and then centrifuged for 5 min at 10,000 × g. One mL of the supernatant was mixed with 2.5 mL of 0.5% thiobarbituric acid in 20% trichloroacetic acid and incubated in hot water (95 °C) for 30 min. Subsequently, it was rapidly cooled on ice to halt the reaction and centrifuged at 10,000 × g for 30 min. Absorbance at 532 and 600 nm was measured, and the MDA concentration was estimated by subtracting the non-specific absorption at 600 nm from the absorption at 532 nm, employing an absorbance coefficient of extinction (155 mM^− 1^ cm^− 1^) (Heath and Packer 1968).

For the determination of H_2_O_2_ content, the procedure outlined by Velikova et al. (2000) was utilized (Velikova et al. 2000).

### Total phenolic compounds

The content of phenolic was determined using folin-ciocalteu reagent with gallic acid as the standard phenolic compound. In a nutshell, 1 g of freeze-dried leaf samples was placed in an eppendorf tube, along with 1 mL of 80% methanol. The mixture was ground at 4 °C and then centrifuged at 10,000 × g for 15 min. The resulting extract was combined with 0.5 mL of folin-ciocalteu reagent (diluted 1:1 with water), and 1 ml of a 5% sodium carbonate solution was added as well. After a 30-minute incubation, absorbance was measured at 725 (Bonyanpour and Jamali 2020).

### Anthocyanins concentration

The measurement of total leaf anthocyanins was carried out spectrophotometrically using the pH differential method employing two buffer systems: a potassium chloride buffer at pH 1.0 (0.025 M) and a sodium acetate buffer at pH 4.5 (0.4 M). For the extraction, 0.5 g of leaf samples were treated with 2 ml of a methanol: water: concentrated HCl solution (80:20:1 v/v/v). Subsequently, 0.4 ml of the leaf extract was mixed with 3.6 ml of the corresponding buffers and the absorbance was recorded against water as a blank at 510 and 700 nm. The absorbance (A) was calculated as follows:

A = (A515 - A700) pH 1.0 - (A510 - A700) pH 4.5.

Then total anthocyanins content was calculated using the equation: Anthocyanin (µg. g^− 1^ fresh weight) = (A × Mw × DF × 1000) / e where A is the absorbance of the diluted sample and DF is the dilution factor (Boonyanitipong et al. 2011), Mw is molecular weight of cyanidin-3-glucoside (449.2) and e = 26,900 L/mol.cm, molar extinction coefficient of cyanidin-3- glucoside (Bonyanpour and Jamali 2020).

### Glutathione (GSH) content

The determination of GSH followed the method established by Moron et al. (1979). Two hundred mg of leaf tissue were homogenized in 2 ml of ice-cold 5% trichloroacetic acid. The homogenate was subsequently centrifuged at 4 °C at 15,000 × g for 30 min. A volume of 75 µl of the clear supernatant was added to a cuvette containing 300 µl of phosphate buffer (0.2 M, pH 8.0) and 750 µl of 0.6 mM DTNB (5,5-dithiobis-2-nitrobenzoic acid) in phosphate buffer. The absorbance at 412 nm was measured, and the GSH content was determined by referring to a standard curve prepared with known amounts of GSH in 5% trichloroacetic acid (Moron et al. 1979).

### Ascorbate content

The concentration of ascorbic acid in the leaf was determined using the Omaye et al. (1979) method. Briefly, 1 g of leaf sample was mixed with 10% ice-cold TCA and centrifuged for 20 min at 3500 × g at room temperature. Subsequently, 1 ml of the supernatant was combined with 0.2 ml of DTC (2,4-dinitrophenyl hydrazine–thiourea–CuSo_4_ reagent) reagent and incubated for 3 h at 37 °C. Afterward, 1.5 ml of ice-cold 65% H_2_SO_4_ was added, and thoroughly mixed, and the solutions were left at room temperature for an additional 30 min. The resulting color was measured spectrophotometrically at 520 nm (Omaye et al. 1979).

### Proline content

The proline content was assessed by following the Bates et al. (1973) method. Leaf segments were first homogenized using a 3% sulfosalicylic acid solution, and the resulting homogenate underwent a 20-minute centrifugation at 3000 × g. The supernatant was then processed with acetic acid and acid ninhydrin, boiled for one hour, and the absorbance was measured at 520 nm (Bates et al. 1973).

### Enzymes extraction

To extract enzymes, 0.5 g of leaves were initially ground into a fine powder using liquid nitrogen and a mortar and pestle. Subsequently, the powdered material was homogenized in 2 ml of extraction buffer, which consisted of 50 mM potassium phosphate buffer at pH 8.0, 10% (w/v) polyvinylpyrrolidone (PVP), 0.1 mM ethylenediaminetetraacetic acid (EDTA), and 1 mM dithiothreitol (DTT). The resulting homogenate was then subjected to centrifugation at 15,000 × g, maintained at 4 °C, for a duration of 30 min. Following centrifugation, the supernatants were collected for further analysis.

### Superoxide dismutase (SOD)

SOD activity was assessed using the following Dhindsa et al. (1980) procedure. To a test tube, 0.1 mL of the enzymatic extract was added, along with a mixture containing 13 mM L-methionine, 25 mM nitroblue tetrazolium chloride (NBT), 0.1 mM EDTA, 50 mM sodium carbonate, and 2 mM riboflavin in a 50 mM phosphate buffer at pH 7.8. The tube was then exposed to two 15 W fluorescent lamps for a duration of 15 min. A control sample, which contained the complete reaction mixture without enzyme and produced the maximum color, was included. The reaction was halted by turning off the lights and placing the tubes in the dark. A non-irradiated complete reaction mixture was used as a blank. The absorbance was measured at 560 nm, and one unit of enzyme activity was defined as the amount of enzyme required to reduce the absorbance reading to 50% in comparison to tubes lacking the enzyme. SOD activity was expressed as units per mg of protein (Dhindsa et al. 1981).

### Catalase(CAT)

CAT activity was determined spectrophotometrically following the method outlined by Chance and Maehly (1955). This method relies on monitoring the decrease in absorbance at 240 nm, which is a result of H_2_O_2_ consumption. In a reaction mixture, one ml consisted of 50 mM potassium phosphate buffer with a pH of 7.0 and 15 mM H_2_O_2_. The reaction was initiated by adding 50 µL of the crude extract to this solution. CAT activity was expressed as units, which indicate the number of micromoles (µmol) of H_2_O_2_ consumed per minute, per milligram of protein (Chance and Maehly 1955).

### Ascorbate peroxidase(APX)

The activity of APX was assessed spectrophotometrically in accordance with the method described by Nakano and Asada (1981). This method relies on monitoring the decrease in absorbance at 290 nm, which is attributed to the oxidation of ascorbate. The rate of ascorbate oxidation was determined within a time frame of 1 to 60 s after initiating the reaction with the addition of H_2_O_2_. In a reaction mixture, one ml consisted of 50 mM potassium phosphate buffer with a pH of 7.0, 0.5 mM ascorbate, 0.15 mM H_2_O_2_, 0.1 mM EDTA, and 50 µL of enzyme extract. APX activity was expressed as units, representing the number of micromoles (µmol) of ascorbate oxidized per minute, per milligram of protein (Nakano and Asada 1981).

### Phenylalanine Ammonia-lyase (PAL)

300 mg of fresh leaf tissue was ground into a fine powder with 6.5 ml of Tris-HCl buffer (50 mM) containing 2-Mercaptoethanol (15 mM) in a chilled mortar. The resulting extract was centrifuged at 5000 g for 30 min. The supernatant obtained was used for the assessment of enzyme activity.

PAL is an enzyme that catalyzes the conversion of phenylalanine to cinnamic acid. PAL enzyme activity is determined by measuring the rate of cinnamic acid formation. One ml of the extraction buffer was employed, consisting of 0.5 ml of L-phenylalanine (10 mM), 1 ml of the enzyme extract, and 0.4 ml of water. This mixture was incubated at 37 °C for 60 min, and the reaction was stopped by adding 0.5 ml of 10% trichloroacetic acid. The absorbance of the solutions was recorded at a wavelength of 290 nm, and the cinnamic acid concentration was determined using the molar absorption coefficient (M^− 1^cm^− 1^) of 9500 (Hahlbrock and Ragg 1975).

### Silver content

Plant roots and leaves were meticulously gathered. To remove silver nanoparticles adhering to the root surfaces, the samples were thoroughly washed. Following this, the samples were dried in an oven at 70 °C for 72 h and then digested in nitric acid. Subsequently, the samples were analyzed using an Agilent 7500a Inductively coupled plasma mass spectrometry (ICP-MS) instrument (USA).

### Statistical analysis

Data were analyzed by spss and means were compared using duncan’s multiple range tests at 5% probability level.

## Results

### Shoot and root length

**Fig. 3:**
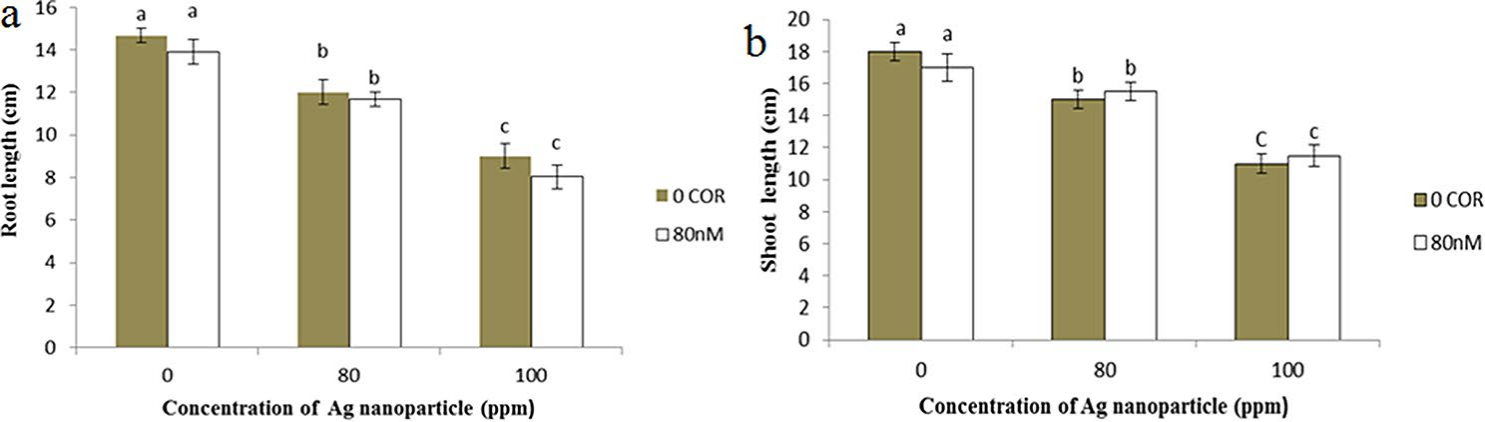
Impact of silver nanoparticles and coronatine on root length (**a**) and shoot length (**b**). Columns with the same letters are not statistically different at 5% probability using duncan’s test. Vertical bars indicate standard errors (*n* = 3)

The influence of coronatine pre-treatment on the root and shoot length of plants exposed to silver nanoparticles is depicted in Fig. 3. Coronatine application, alone, did not have any significant impact on either shoot or root length (Fig. 3a, b). However, when silver nanoparticles were used independently, they resulted in a reduction in both of these characteristics compared to the control group. At concentrations of 80 and 100 ppm, silver nanoparticles led to an 18% and 38% decrease in root length, respectively, in comparison to the control group. Furthermore, shoot length exhibited a reduction of 16% and 38% at the same silver nanoparticle concentrations (80 and 100 ppm), respectively, in comparison to the control.

### Chlorophyll and carotenoids

**Fig. 4:**
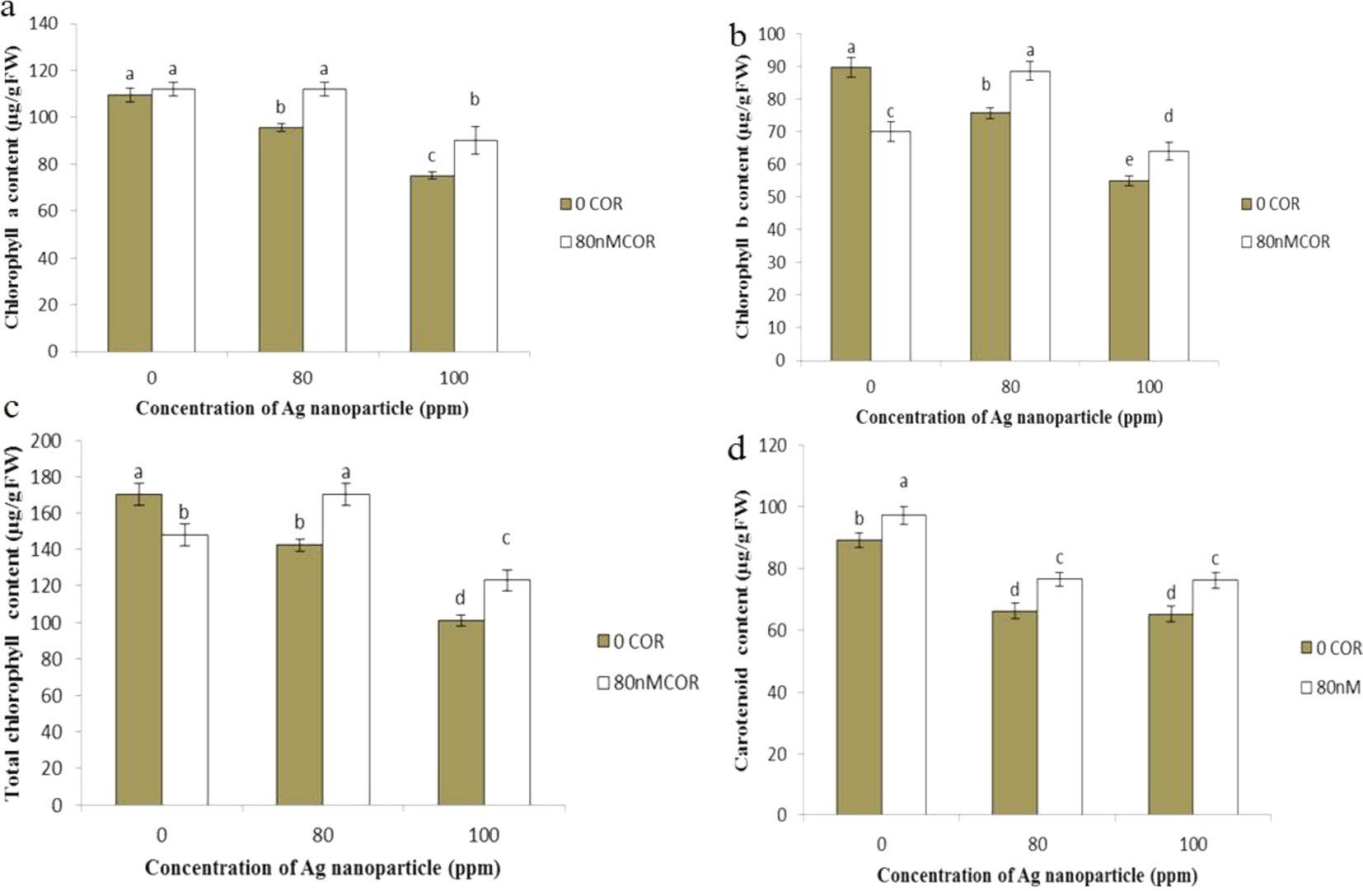
- Effect of silver nanoparticles and coronatine on leaf chlorophyll a (**a**), chlorophyll b (**b**), total chlorophyll (**c**), and carotenoids (**d**). Columns with the same letters are not statistically different at 5% probability using duncan’s test. Vertical bars indicate standard errors (*n* = 3)

When applied alone, coronatine didn’t significantly affect the content of chlorophyll a but did lead to a reduction in chlorophyll b, and total chlorophyll levels compared to the control group (Fig. 4a, b). On the other hand, silver nanoparticles, when used independently and at increasing concentrations, caused a decrease in the levels of chlorophyll a, chlorophyll b, total chlorophyll, and carotenoids compared to the control group.

When coronatine and silver nanoparticles were combined, it resulted in an increase in the content of chlorophyll a, chlorophyll b, total chlorophyll, and carotenoids compared to the stress induced by silver nanoparticles, thereby mitigating the adverse effects of silver nanoparticles. The lowest levels of chlorophyll a, chlorophyll b, and total chlorophyll were observed when plants were exposed to silver nanoparticles (100 ppm), while the highest levels were found in the control and plants treated with coronatine (80 nM) and exposed to silver nanoparticles at a concentration of 80 ppm (Fig. 4).

### Anthocyanins and total phenolic compounds

Total phenolic compound concentration increased significantly when plants were treated with coronatine alone. On the other hand, exposure of plants to silver nanoparticles, resulted in a reduction of 24% at concentration 100 ppm, compared to the control.

**Fig. 5:**
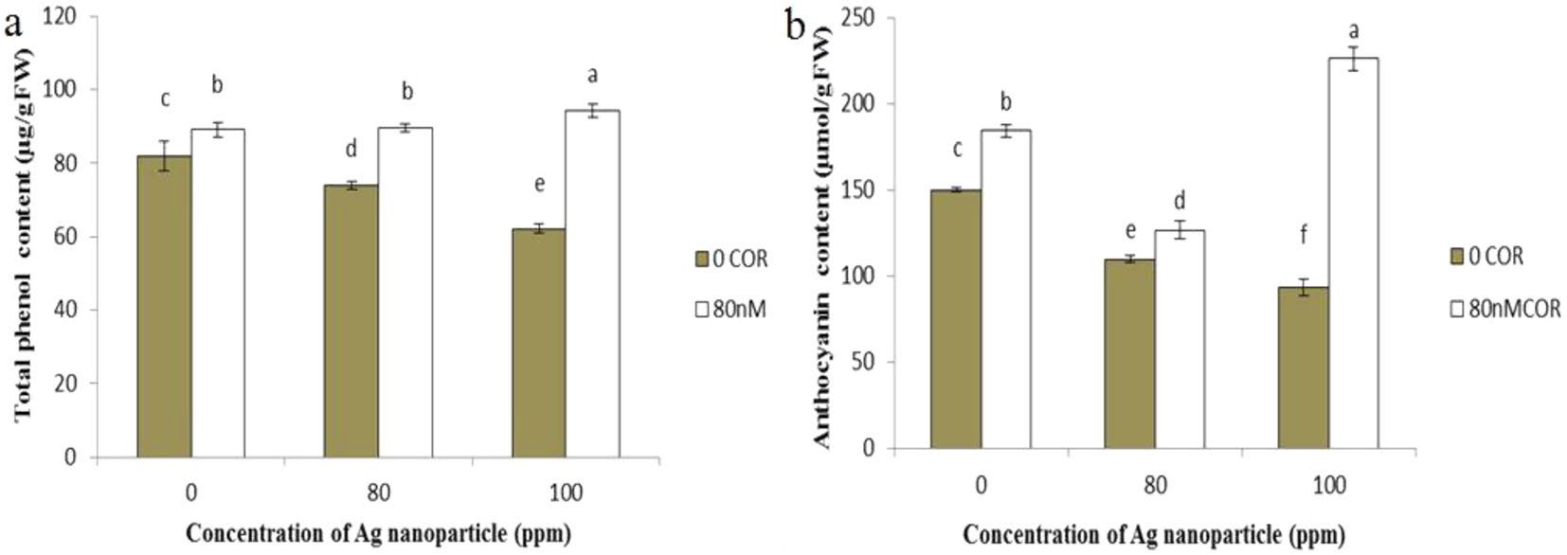
Effect of silver nanoparticles and coronatine on leaf total phenolic content (**a**) and anthocyanin content (**b**). Columns with the same letters are not statistically different at 5% probability using duncan’s test. Vertical bars indicate standard errors (*n* = 3)

However, when coronatine treatment was combined with silver nanoparticles, it led to an increase in the total phenolics content compared to plants exposed to silver nanoparticles alone (Fig. 5a).

As illustrated in Fig. 5b, the anthocyanin content increased when plants were treated with coronatine, but treatment with silver nanoparticles alone, at concentrations of 80 and 100 ppm, caused a decrease in anthocyanin content. The combination of coronatine with silver nanoparticles resulted in an increase in anthocyanin content compared to exposure of plants to silver nanoparticles alone.

#### MDA and H_2_O_2_ content

**Fig. 6:**
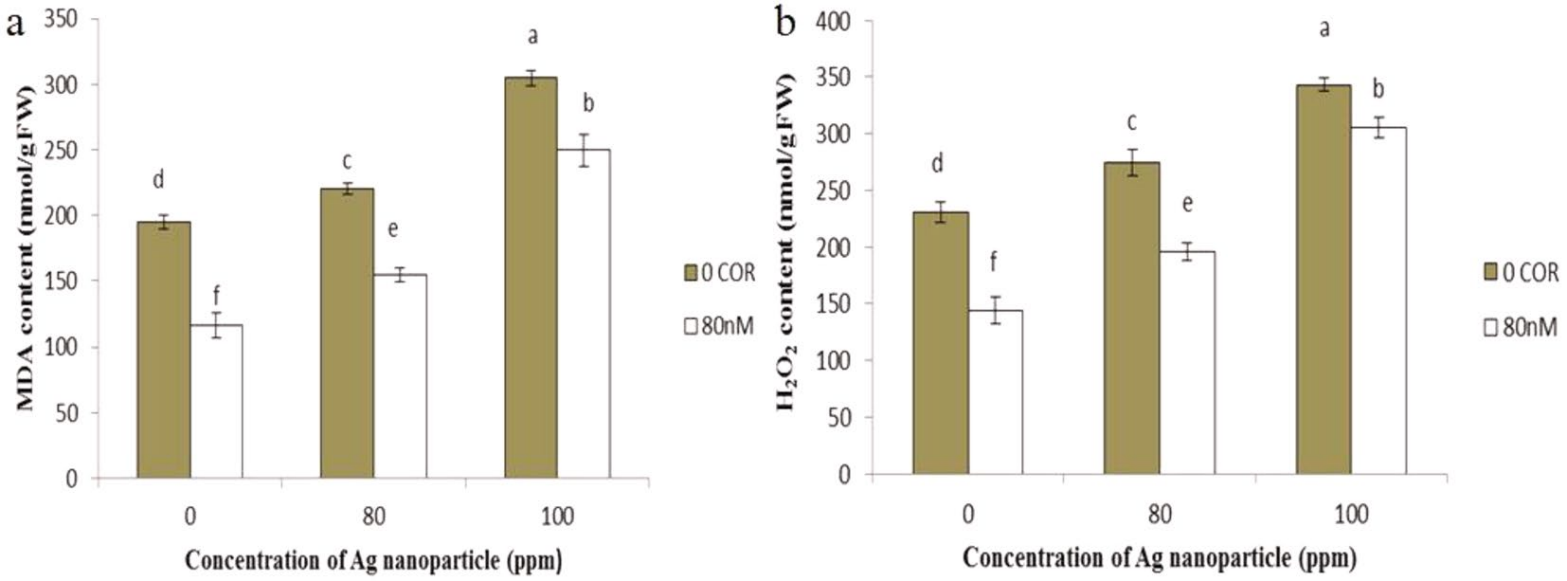
The impact of silver nanoparticles and coronatine on MDA content (**a**) and H_2_O_2_ content (**b**). Columns with the same letters are not statistically different at 5% probability using duncan’s test. Vertical bars indicate standard errors (*n* = 3)

When applied individually, coronatine led to a decrease in both MDA and H_2_O_2_ content compared to the control group. However, as the nanoparticle concentration increased, the levels of MDA and H_2_O_2_ increased in comparison to control. Conversely, when coronatine was applied in conjunction with silver nanoparticles, it resulted in a reduction of MDA and H_2_O_2_ content compared to plants exposed to silver nanoparticles alone, thus effectively mitigating their adverse effects. The highest MDA and H_2_O_2_ levels were observed in the samples exposed to 100 ppm silver nanoparticles, while the lowest levels were found in plants treated with coronatine alone (Fig. 6a, b).

### Ascorbate and GSH content

**Fig. 7:**
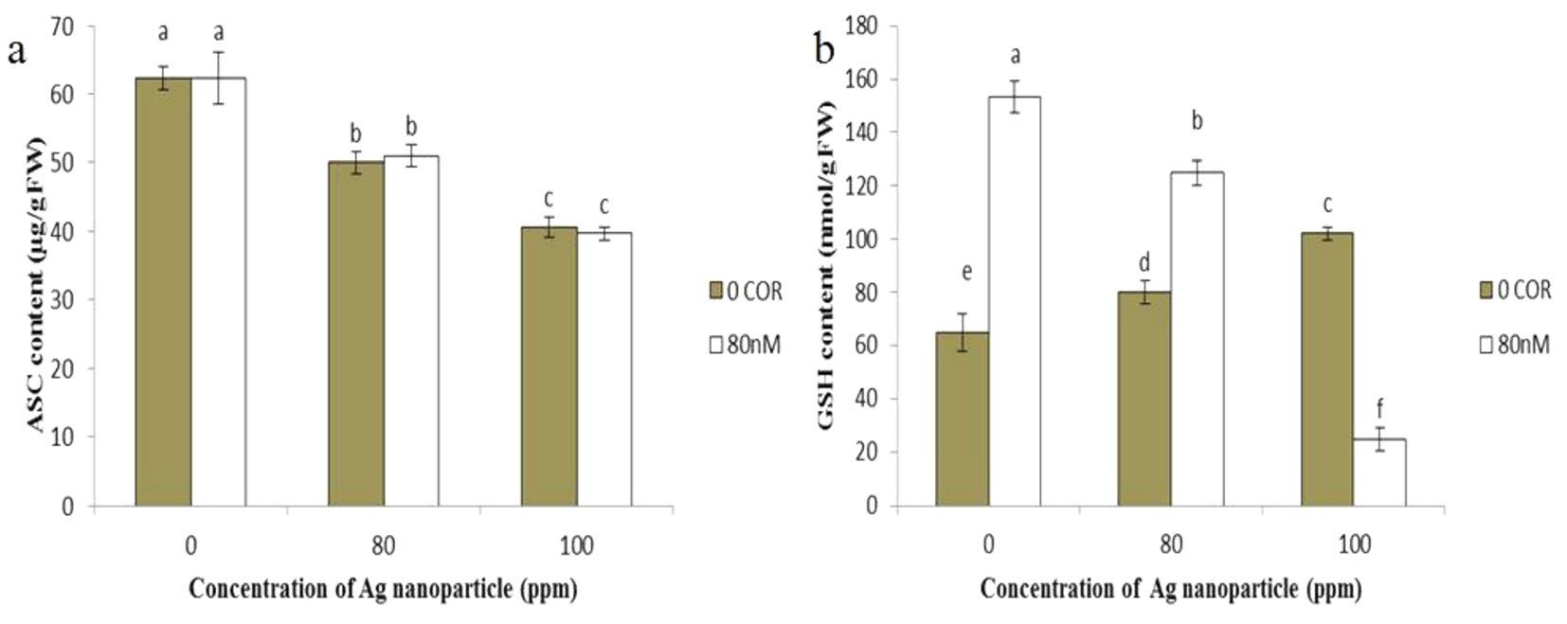
Effect of silver nanoparticles and coronatine on ascorbate content (**a**) and GSH content (**b**). Columns with the same letters are not statistically different at 5% probability using duncan’s test. Vertical bars indicate standard errors (*n* = 3)

Coronatine, when applied individually, did not significantly alter the ascorbate content compared to the control group. However, when plants were exposed to silver nanoparticles, there was a 19% and 34% reduction compared to the control at concentrations of 80 and 100 ppm, respectively. When coronatine was combined with silver nanoparticles, no significant changes were observed compared to treatment with silver nanoparticles alone (Fig. 7a).

The GSH content increased when plants were treated with coronatine compared to the control. Exposure of plants to silver nanoparticles alone, at concentrations of 80 and 100 ppm, resulted in an increase in GSH content compared to the control (Fig. 7b). When silver nanoparticle-exposed plants were treated with coronatine, it resulted in an increase in GSH content at the 80 ppm concentration of silver nanoparticles and a decrease at the 100 ppm concentration compared to treatment with silver nanoparticles at the same concentrations without the application of coronatine.

#### Proline concentration

When applied individually, coronatine showed no significant effect on the proline content compared to the control group. In contrast, the use of silver nanoparticles alone at a concentration of 100 ppm led to a reduction in proline content compared to the control group, while no significant changes were observed at a concentration of 80 ppm.

**Fig. 8:**
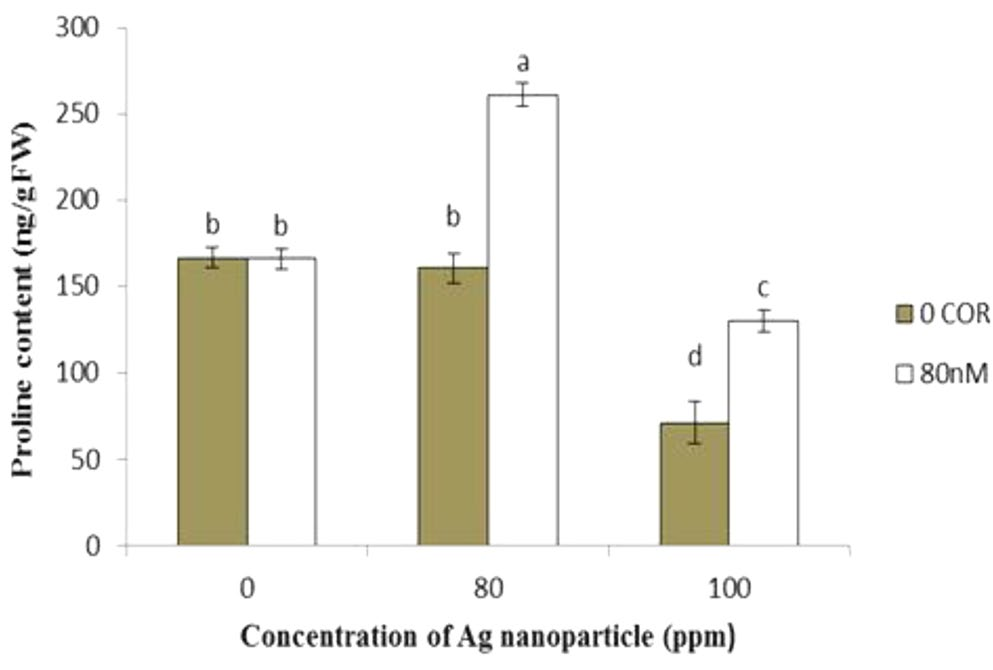
Effect of silver nanoparticles and coronatine on proline content. Columns with the same letters are not statistically different at 5% probability using duncan’s test. Vertical bars indicate standard errors (*n* = 3)

The combination of coronatine with silver nanoparticles led to an increase in proline content compared to plants exposed to silver nanoparticles alone. The lowest proline content was observed in plants treated with 100 ppm silver nanoparticles, while the highest proline content was observed in plants treated with 80 nM coronatine + 80 ppm silver nanoparticles (Fig. 8).

#### Enzymatic activity

**Fig. 9:**
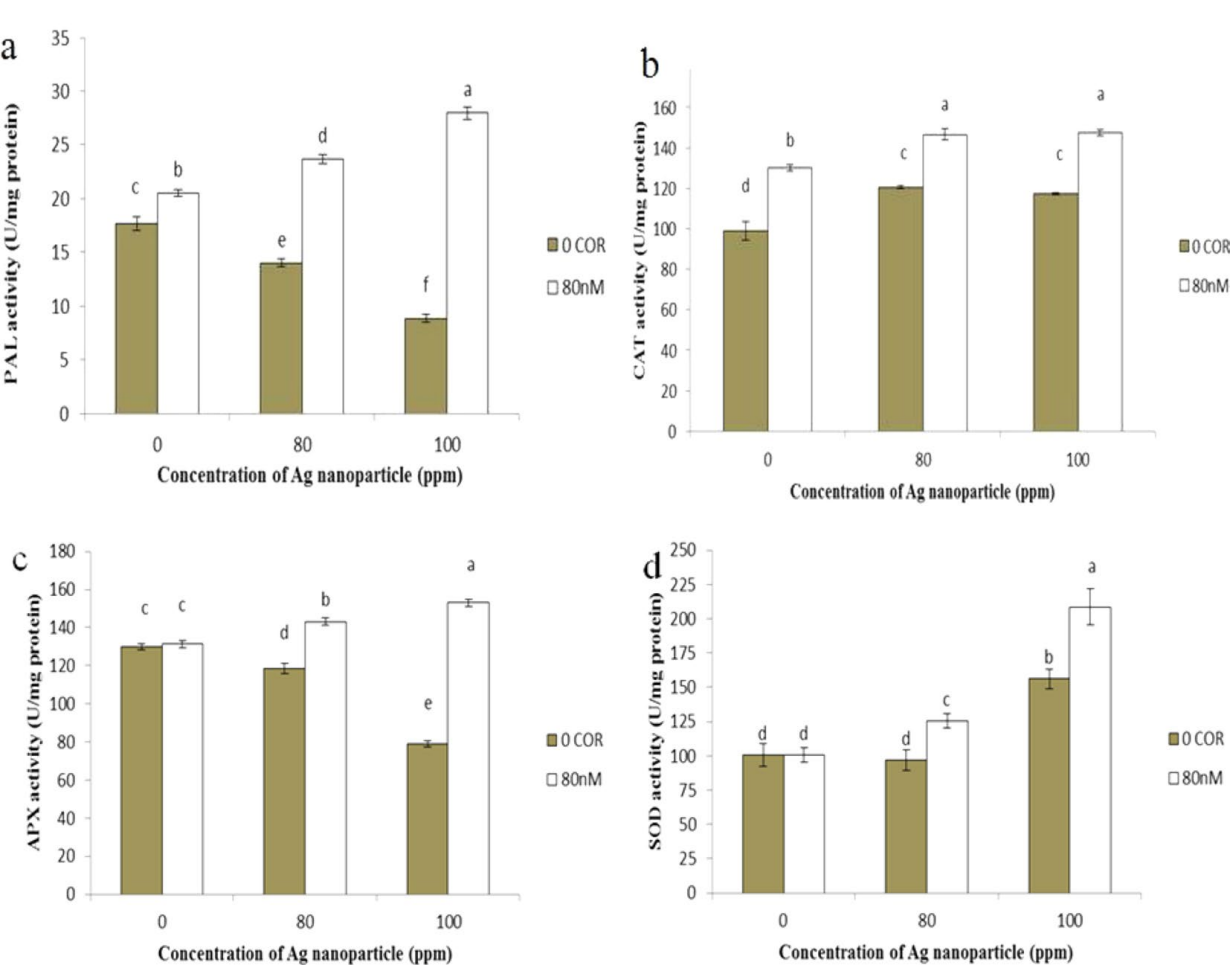
The impact of silver nanoparticles and coronatine on PAL (**a**), CAT (**b**), APX (**c**), and SOD (**d**) enzymatic activities. Columns with the same letters are not statistically different at 5% probability using duncan’s test. Vertical bars indicate standard errors (*n* = 3)

The enzyme activities of CAT and PAL increased significantly with coronatine pre-treatment, while the activities of SOD and APX remained relatively stable compared to the control group. Exposure of plants to silver nanoparticles induced an increase in the activities of SOD and CAT, in contrast to a decrease in APX and PAL activities when compared to the control. When coronatine was combined with silver nanoparticles, it led to an overall increase in the activities of all enzymes compared to plants exposed to silver nanoparticles alone (Fig. 9).

#### Silver concentration

**Fig. 10:**
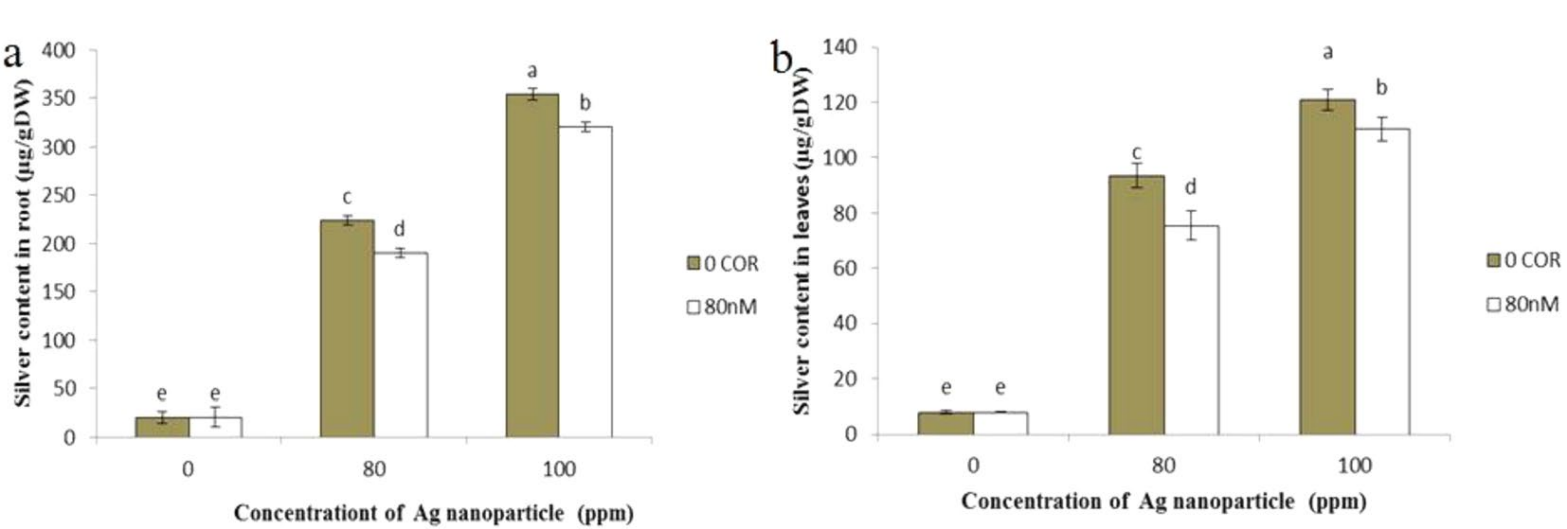
The impact of silver nanoparticles and coronatine on root silver content (**a**) and shoot silver content (**b**). Columns with the same letters are not statistically different at 5% probability using duncan’s test. Vertical bars indicate standard errors (*n* = 3)

Figure 10 illustrates the silver concentration within both the roots and the shoots of the plants. Coronatine had no significant effect on this parameter compared to the control group. Parallel to an increased concentration of silver nanoparticles, there was a corresponding increase in the silver content. However, pre-treatment with coronatine led to a decrease in silver content in both roots and shoots compared to plants exposed to silver nanoparticles without coronatine treatment. The highest silver content in both roots and shoots was observed in plants exposed to a concentration of 100 ppm silver nanoparticles.

## Discussion

Silver nanoparticles, when applied individually, caused a reduction in root and shoot length, while coronatine, when applied independently, had no effect on root and shoot length. The growth inhibition in response to exposure to silver nanoparticles could be due to increased production of reactive oxygen species (ROS). ROS, including superoxide, singlet oxygen, hydroxyl radicals, and H_2_O_2_, cause extensive oxidative damage by disrupting membrane structures and affecting essential macromolecules in cellular structures, such as proteins, lipids, pigments, and nucleic acids (Sahu et al. 2022).

Several studies suggest that the harmful effects of silver nanoparticles on plants may be caused by: (i) excessive production of ROS by redox cycling; (ii) size dependent mechanical damage of nanoparticles (Jiravova et al. 2016; Amooaghaie et al. 2018); (iii) oxidation of silver nanoparticles and produced silver ions (Shahraki et al. 2024).

Consequently, the adverse impacts of silver nanoparticles on the growth of cress plants in our study could be attributed to the induction of oxidative stress, silver accumulation, and their influence on photosynthetic activities.

According to our results the application of silver nanoparticles led to a reduction in the concentration of chlorophyll a, b, total chlorophyll, and carotenoids. The decrease in chlorophyll content may attributed to the effects of silver ions released by the nanoparticles on the enzymatic pathways involved in chlorophyll biosynthesis, which could lead to a substitution of magnesium in the chlorophyll structure (*52*).

In addition, silver nanoparticles can influence photosynthesis by competitively displacing copper in plastocyanin. In this way, the silver ions released from the nanoparticles bind to plastocyanin and compete with copper for binding sites. The substitution of silver in plastoquinone leads to a disruption of the photosynthetic electron transfer process and ROS production (Jansson and Hansson 2008; Sujak 2005).

While adverse effects of silver nanoparticles on chlorophyll content have been reported more frequently, there are also cases where silver nanoparticles were able to stimulate chlorophyll production. Jowkar et al. (2013) observed a significant increase in chlorophyll content in all samples exposed to silver nanoparticle treatment (Jowkar et al. 2013). Bagherzadeh Homaee et al. (2015) reported that the chlorophyll content of leaves increased significantly at low concentrations of silver nanoparticles and decreased at higher concentrations of this treatment (Bagherzadeh Homaee and Ehsanpour 2015).

In this study, the adverse effects of silver nanoparticles on the reduction of leaf pigment content were ameliorated by coronotine pretreatment. Wang et al. (2008) suggested that coronatine has the potential to alleviate oxidative stress, leading to an increase in chlorophyll content and improved membrane stability in maize seedlings under drought stress conditions (Wang et al. 2009).

The integrity of the cell membrane is a very important indicator that can be impaired by stress (Tripathi et al. 2017; Nair and Chung 2014a, b). ROS generated during stress can react with polyunsaturated fatty acids in the cell membrane, leading to increased lipid peroxidation. A byproduct of lipid peroxidation in the plasma membrane is MDA. In the present study, H_2_O_2_ production increased upon treatment with silver nanoparticles, which led to an increase in MDA production due to the effect on lipid membranes. Pretreatment with coronatine improved the toxicity of silver nanoparticles by reducing the H_2_O_2_ content. The present findings are in agreement with the results of Zare Dehabadi et al. (2013), who indicated that exogenous application of coronatine reduced MDA overproduction in sweet basil seedlings under arsenic stress (Zare Dehabadi et al. 2013).

To protect important molecular and cellular structures from the damaging effects of ROS, a complex network of antioxidant defense mechanisms is activated in plant cells (Rico et al. 2015).

Plants possess antioxidant defense systems to counteract the damaging effects of ROS. These defense mechanisms encompass both non-enzymatic and enzymatic antioxidant systems. Non-enzymatic antioxidants include thiols, ascorbate, glutathione, phenols, and anthocyanins, while enzymatic antioxidants encompass APX, CAT, and SOD.

Compatible solutes, such as proline, play a pivotal role as non-enzymatic antioxidants in scavenging ROS (Chen and Murata 2002). Proline also contributes to osmoregulation, restoration of chlorophyll structure, protection of enzymes from denaturation, and the stabilization of macromolecules and organelles.

In the present study, a decrease in proline content was observed in cress plants exposed to silver nanoparticles, while coronatine treatment mitigated this reduction in proline content induced by silver nanoparticles. Our findings are consistent with the increase in proline observed with coronatine treatment in sweet basil plants under arsenic stress (Zare Dehabadi et al. 2013).

In any case, the increased proline in plants pre-treated with coronatine can help to reduce oxidative stress and improve membrane integrity.

Phenolic compounds, including flavonoids, anthocyanins, tannins, hydroxycinnamic esters, and lignins, are a group of secondary metabolites derived from the phenylpropanoid pathway (Crozier et al. 2006). They are commonly found in plant tissues, and while they are naturally synthesized in plant cells, their levels can be influenced by environmental stressors and prevailing conditions. These fluctuations are often linked to changes in the activity of enzymes involved in both the synthesis and degradation of these compounds, thereby affecting their concentration within plant cells. Numerous studies on plants have demonstrated that abiotic stresses like exposure to nanoparticles and heavy metal can significantly impact the levels of phenolic compounds, phytochelatins, anthocyanins, and the activity of the PAL (Goncharuk and Zagoskina 2023; Jańczak-Pieniążek et al. 2022; Krishnaraj et al. 2012).

In our study, the concentration of anthocyanins and phenolic content in cress plants decreased when exposed to silver nanoparticles at concentrations of 80 and 100 ppm, while treatment with coronatine reduced the silver nanoparticle-induced decrease in anthocyanin and phenolic content. These findings are consistent with previous studies indicating a decrease in the content of phenolic compounds in lettuce seedlings when treated with silver nanoparticles (Kalisz et al. 2021).

The reduction in anthocyanin and phenolic content may result from a diversion of precursor molecules within the their synthesis pathway or a change in oxidative stress level, which in turn affects the activity of enzymes (Moazzami Farida et al. 2020).

These compounds share cinnamic acid as their precursor, and cinnamic acid is a product of PAL enzyme activity (on phenylalanine), thus changes in PAL activity may contribute to variations in the levels of these compounds within plants. PAL represents the principal enzyme in the phenylpropanoid pathway, catalyzing the deamination conversion of L-phenylalanine into trans-cinnamic acid. Cinnamic acid serves as the primary precursor for the synthesis of phenolic compounds, anthocyanins, flavonoids, tannins, and lignins (Prakash et al. 2007). Alterations in PAL activity and other enzymes within the phenylpropanoid pathway, as well as the accumulation of various phenolic compounds, may constitute one of the initial responses to abiotic stresses. Consequently, changes in PAL activity and expression appear to be integral to plants’ initial reactions when exposed to various a/biotic stressors (Aghdam et al. 2012).

In the present study, PAL activity decreased in plants exposed to silver nanoparticles, whereas treatment with coronatine resulted in an increase in PAL activity in plants exposed to silver nanoparticles. In agreement with our results, PAL activity reduced in wheat plants exposed to silver nanoparticles. (Bano and Ummat ul 2020).

The decrease in PAL enzyme activity exposed to silver nanoparticles can be due to the decrease in substrate or phenylalanine amino acid. Yang et al. (2018) reported a decrease in phenylalanine content in wheat due to silver nanoparticles (Yang et al. 2018). In addition to the enzymatic antioxidant system, the non-enzymatic antioxidant system also plays an important role in neutralizing the effects of ROS (Das and Roychoudhury 2014; Gould and Nature’s Swiss Army Knife 2004).

APX and CAT enzymes are homoproteins responsible for scavenging and neutralizing ROS and for maintaining a delicate equilibrium between ROS production and degradation within plant cells. Various abiotic stressors are implicated in causing oxidative stress, leading to alterations in the activities of antioxidant enzymes in plants (Caverzan et al. 2016). The results of this study demonstrated that the activity of antioxidant enzymes, such as SOD, APX, and CAT, changed when plants were exposed to silver nanoparticles.

Enzymes such as SOD and CAT, working in synergy with the enzymes of the ascorbate-GSH cycle, are important components of a plant’s antioxidant defense system. Remarkably, GSH levels increased in response to silver nanoparticles, while ascorbate levels decreased. Similarly, increased upregulation of genes involved in GSH biosynthesis, GSH-S-transferase, and GSH reductase was observed after Arabidopsis plants were exposed to silver nanoparticles (Nair and Chung 2014a, b). In contrast, *Rosmarinus officinalis* exhibited an increase in ascorbic acid content when subjected to silver nanoparticle treatment (Hadi Soltanabad et al. 2020).

The SOD enzyme is the first line of defense against ROS, effectively neutralizing superoxide ions (O_2_^−^) and converting them into H_2_O_2_ and molecular oxygen. CAT and APX enzymes are responsible for detoxifying any excess H_2_O_2_ within plant tissues (Tripathi et al. 2017). APX uses ascorbate as an electron donor to detoxify H_2_O_2_, while CAT performs this function independently of external reducing agents. In our research, exposure of plants to silver nanoparticles resulted in an increase in the activity of SOD, CAT enzymes compared to the control group, while APX enzyme activity decreased. The reduction in APX enzyme activity in plants exposed to silver nanoparticles could be attributed to a decrease in ascorbate content as an electron donor, potentially coupled with damage to the enzyme caused by the toxic effects of silver (Ziotti et al. 2021).

Simultaneous treatment with coronatine and silver nanoparticles resulted in increased activity of all enzymes compared to nanoparticle stress. Changes in the content of antioxidant molecules and in the activity of enzymatic antioxidants are signals for improved tolerance and adaptation to oxidative stress conditions.

In agreement with our findings, studies on the impact of silver nanoparticles on *Arabidopsis thaliana* showed an increase in SOD activity (Nair and Chung 2015). Additionally, Cecilia Barrios et al. (2016) found that various concentrations of cerium oxide nanoparticles had no significant effect on CAT enzyme activity compared to the control, but they had a decreasing effect on APX enzyme activity (Barrios et al. 2016). Despite studies on enzyme activities in different plants exposed to nanoparticles, the effects have been shown to be erratic and unpredictable. For instance, titanium dioxide nanoparticles increased the activities of SOD, CAT, and APX in spinach (Lei et al. 2008), while they decreased the activities of APX in *Vicia faba* (Foltête et al. 2011). Zhao et al. (2012) reported that APX activity increased in maize exposed to cerium oxide nanoparticles (Zhao et al. 2012).

The physiological impacts and toxicity of nanoparticles in plants were closely linked to the level of silver accumulation within the plant. The presence of nanoparticles resulted in an increase in silver content in shoots and roots. Simultaneous treatment with coronatine and nanoparticles led to a reduction in silver content, potentially playing a role in enhancing the plant’s tolerance to nanoparticle toxicity. The effects of coronatine in reducing silver concentration can be attributed to its role in increasing the phenol content in plants. The reduction of silver concentration in the plant can be influenced by the accumulation or synthesis of compounds such as phenolic compounds. It has been reported that the absorption of nickel and cadmium in chamomile plants was significantly inhibited by the strong accumulation of phenolic compounds (Kovácik et al. 2011).

However, the exact mechanism underlying the reduction in silver content in plants treated with coronatine remains unclear and therefore requires further investigation.

## Conclusion

**Fig. 11:**
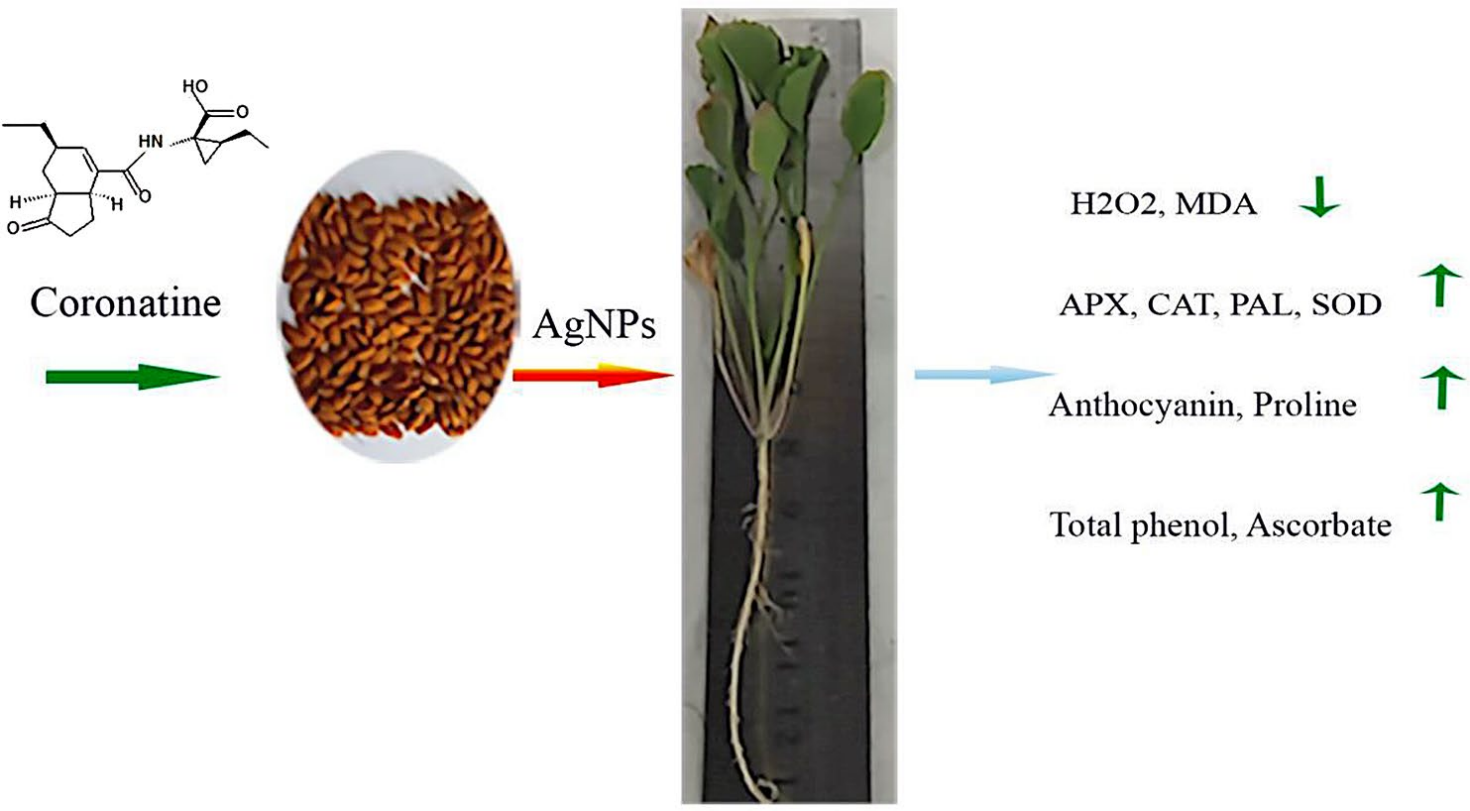
- The alleviating effects of coronatine on silver nanoparticle stress in cress plant

In summary, our results show that silver nanoparticles induce oxidative stress in cress plants, leading to reduced plant growth and leaf pigment content. The application of coronatine increases tolerance to nanoparticles by regulating silver accumulation and strengthening the plant’s antioxidant defense system. Our results suggest that coronatine could be a promising treatment to mitigate the toxicity of nanoparticles in crop production. The beneficial effects of alleviating silver nanoparticle stress in cress plants by coronatine are summarized in Fig. 11.

## Abbreviations

MDA: Malondialdehyde
CAT: Catalase
SOD: Superoxide dismutase
APX: Ascorbate peroxidase
GSH: Glutathione
DTC: 2,4-dinitrophenyl hydrazine–thiourea–CuSO_4_ reagent
PAL: Phenylalanine ammonia-lyase
ROS: Reactive oxygen species
